# Updating the Good Reporting of a Mixed Methods Study (GRAMMS) Reporting Guidelines: Protocol for a Methodological Review and Modified Delphi Process

**DOI:** 10.2196/82364

**Published:** 2026-03-17

**Authors:** Sarah Munce, Sergi Fàbregues, Quan Nha Hong, Alicia O’Cathain, Timothy Guetterman, John Creswell, Cheryl Poth, Katie Dainty, Andrea C Tricco, Mandy M Archibald, Clementine Jarrett, Jennifer Boyle, Dorothy Luong, Frank Kiwanuka, Ahtisham Younas

**Affiliations:** 1 Holland Bloorview Kids Rehabilitation Hospital Bloorview Research Institute Toronto, ON Canada; 2 Department of Psychology and Education Universitat Oberta de Catalunya Barcelona Spain; 3 School of Rehabilitation Faculty of Medicine Université de Montréal Montréal, QC Canada; 4 Centre for Interdisciplinary Research in Rehabilitation of Greater Montreal Montréal, QC Canada; 5 School of Medicine and Population Health University of Sheffield South Yorkshire United Kingdom; 6 Department of Family Medicine University of Michigan Ann Arbor, MI United States; 7 Faculty of Education University of Alberta Edmonton, AB Canada; 8 Research and Innovation North York General Hospital Toronto, ON Canada; 9 Li Ka Shing Knowledge Institute St. Michael’s Hospital Toronto, ON Canada; 10 Institute of Health Policy, Management, and Evaluation University of Toronto Toronto, ON Canada; 11 College of Nursing University of Manitoba Winnipeg, MB Canada; 12 Children’s Hospital Research Institute of Manitoba University of Manitoba Winnipeg, MB Canada; 13 Carleton University Ottawa, ON Canada; 14 Faculty of Health Sciences University of Eastern Finland Kuopio Finland; 15 Memorial University of Newfoundland St. John’s, NL Canada; 16 Biruni University Istanbul Turkey

**Keywords:** mixed-methods research, methodological review, modified delphi process, guideline development, evidence-informed guidelines

## Abstract

**Background:**

In mixed methods research (MMR), researchers combine elements of qualitative and quantitative methodologies, methods of data collection and analysis, viewpoints, and integration procedures to gain a deeper understanding of what is being studied, design culturally specific tools, and explore the conditions under which health care interventions succeed or fail. Integration is considered the hallmark of MMR and can occur at various levels, such as sampling, data collection, and analysis. MMR is particularly useful for investigating complex, multilevel programs and interventions and is well-suited to address research problems involving knowledge translation, program evaluations, or comparisons of therapeutic interventions. Although there are many potential benefits of mixed methods in health research, the extent to which mixed methods studies implement integration remains limited, with this specific gap persisting for almost 20 years. The Good Reporting of a Mixed Methods Study (GRAMMS) reporting guidelines were developed in 2008 to help improve the quality of reporting in mixed methods reports and articles. Since then, the field of mixed methods has evolved rapidly, and the guideline no longer reflects current practices and innovations.

**Objective:**

The objective of this study is to develop an updated GRAMMS 2.0 guideline. This project aims to develop the GRAMMS 2.0 guideline and checklist to improve consistency, transparency, and quality of reporting on studies that use mixed methods in health services research. The specific research objectives of this protocol are to (1) examine the extent of mixed methods methodological literature and identify relevant reporting quality criteria for inclusion in GRAMMS 2.0 (ie, a methodological review), and (2) using the results of the first objective, prioritize the components of the updated GRAMMS 2.0 (ie, modified Delphi approach and consensus meeting).

**Methods:**

This study will follow established methodological frameworks for reporting guideline development and will include a methodological review followed by a modified Delphi process.

**Results:**

The project has received funding from the Canadian Institutes of Health Research in April 2025.

**Conclusions:**

The GRAMMS 2.0 guideline will improve the consistency, transparency, and quality of reporting of mixed methods studies by researchers and multiple knowledge users in policy and practice. Peer reviewers and editors may also use GRAMMS 2.0 to improve the review of manuscripts involving MMR. Ultimately, the updated guideline will increase the clarity of mixed methods research findings, thereby improving their potential transferability to practice and facilitating efficient use of new results in mixed methods in health research, bringing better returns on research investments.

**International Registered Report Identifier (IRRID):**

PRR1-10.2196/82364

## Introduction

Health research often involves investigating complex processes and systems using quantitative (ie, numerical) and qualitative (eg, participant experiences) research approaches [1,2]. The specific research question or objective drives the selection of research methods [3,4]. Quantitative methodologies are often used to address questions related to causality, efficacy, effectiveness, generalizability, size of effects, and prediction. Qualitative methodologies are typically used to understand individual experiences or perspectives, explain why or how a phenomenon occurs, or develop hypotheses or theories [5]. Mixed methods research (MMR) is defined as “the type of research in which researchers combine elements of qualitative and quantitative methodologies, methods of data collection and analysis, viewpoints, and integration procedures to gather viewpoints, breadth and depth of understanding, and corroboration of a particular phenomenon” [6]. Mixed methods research leverages the strengths of both quantitative and qualitative approaches and is an essential methodology for studying the “wicked problems” defined as “those that involve multiple interacting systems, are replete with social and institutional uncertainties, and for which only imperfect knowledge about their nature and solutions exist” [7]*.* Such problems are inherent in health research due to the intricacy of health care systems and service delivery. The explicit mixing or linking of qualitative and quantitative components within a mixed methods study allows researchers to answer questions with greater depth and understanding than possible with one methodology alone [3,4]. This explicit integration is increasingly necessary as health system challenges are becoming more complex, requiring systems-based solutions inclusive of various data types [8]. Mixed methods research is particularly useful for investigating complex, multilevel programs and interventions [3,8,9] and is well-suited to address research problems involving program evaluations, comparisons of therapeutic interventions, or implementation [10,11].

Integration refers to “purposeful interdependence between different sources, methods, approaches” [12]. Integration lies at the heart of MMR [9,13-15], and can occur at multiple levels or dimensions through theory, conceptual models, design, methods, results, analysis, interpretation, visualization, presentation, publication, and teams [15]. Meaningful integration allows researchers to achieve the goal of mixed methods to “produce a whole through integration that is greater than the sum of the individual qualitative and quantitative parts” [16]. For example, qualitative inquiry can be used to provide greater depth to the quantitative findings by integrating qualitative and quantitative results (ie, merging) to examine complementarity and expansion. Quantitative results can also be used to generate the qualitative sample (ie, connecting). Qualitative inquiry can lead to developing or refining quantitative instruments (ie, building) or interventions or generating hypotheses in the qualitative component for testing in the quantitative component [3,5,17,18]. Joint displays, which provide a visual means for implementing and reporting on mixed methods integration, including generating metainferences, are increasingly seen as an area of innovation for advancing integration. However, joint displays in health research remain underused [19-21]. This, along with the emergence of new integrative modalities in MMR (eg, timelines) [22], highlights the imperative for their inclusion in MMR reporting.

Researchers conducting mixed methods studies also need to decide on the timing of the qualitative and quantitative components (ie, convergent or sequential data collection and analysis) and each method’s intent [3,4,23]. For example, Stuber and colleagues [24] used a sequential explanatory design, where the quantitative component was first conducted and was followed by interviews and focus groups (qualitative) to explain initial survey results (quantitative) in a study of patient perceptions of patient-driven care in chiropractic practice. In addition to the mixing or linking of 2 unique research approaches—qualitative and quantitative—mixed methods studies may also involve data transformation (ie, converting qualitative data into quantitative data (“quantitizing”) or vice versa (“qualitizing”) [9]. As such, mixed methods studies can become complex investigations that require additional time and resources and a team of researchers with expertise in quantitative, qualitative, and mixed methodologies, specifically [25].

In 2015, Fetters and Freshwater [16] put forward the 1+1=3 challenge, encouraging health services researchers and others interested in MMR to consider the question*,* “What synergy was gained by the additional work of using both qualitative and quantitative data methods?” The authors urged researchers to plan their studies judiciously with purposeful choices that leverage integration. Although there are many potential benefits of MMR in health services research, the extent to which mixed methods studies provide or report evidence of integration remains limited, with this specific gap in the field persisting for almost 20 years [3,26-28]. This gap remains despite substantial progress in specific areas of integration including integration strategies, assessment of fit of integration, metainferences, and joint displays, as well as other critical aspects, such as methodological quality, reporting of cross-cultural mixed methods studies, intersectional mixed methods, sex/gender-based analysis, considerations of equity and diversity reporting in MMR, and arts-based MMR [3,12,17,18,20,25,29-36]. Moreover, quality guidance and reporting criteria do not reflect the current developments in integration.

Recent reviews of empirical studies in specific fields (eg, primary care, palliative and end-of-life care, and chiropractic services) have examined the mixed methods literature and highlighted areas for improvement in the quality of reporting (ie, whether integration procedures are described and whether evidence of integration is provided) [8,27,33,37-42]. For instance, a review of health services research found that authors of mixed methods studies typically did not describe or justify the need for a mixed methods design or integrate data and findings from the individual quantitative and qualitative components [5,41]. This lack of integration prevents readers from knowing what new insights have been generated by combining quantitative and qualitative components (ie, beyond the results obtained from the two separate components), thereby limiting the methodological potential of this research strategy [5].

The review of health services research formed the basis of the Good Reporting of a Mixed Methods Study (GRAMMS) in 2008 [5]. GRAMMS includes the following components: (1) describe the justification for using a mixed methods approach to the research question; (2) describe the design in terms of purpose, priority, and sequence of methods; (3) describe each method in terms of sampling, data collection, and analysis; (4) describe where integration has occurred, how it has occurred, and who has participated in it; (5) describe any limitation of one method associated with the presence of the other method; and (6) describe any insights gained from mixing or integrating methods. Despite the foundational contribution of the GRAMMS, its application in health research is underused. GRAMMS is more than 15 years old and needs to be updated to better reflect current practices and innovations in the field of MMR. Indeed, the field has advanced substantially [37,43] in specific areas of integration, including integration strategies [3,12,17,18,27,30,31], assessment of fit of integration and meta-inferences [3,25,30,32], joint displays [20,33,34], and other critical aspects, such as methodological quality and reporting [35,37], generalization [36,44], as well as cross-cultural MMR [36]. More critically, some of the GRAMMS criteria require additional detail and attention, especially the criterion related to integration (criterion 4). This criterion does not consider recent advances in this area, and since integration is an essential aspect of an MMR study, more than one criterion on this topic is necessary. Indeed, recently Hirose and Creswell [45] put forward a shortlist of quality criteria in MMR that highlighted the need for much more detail related to integration, reflecting the advances in the field, including mixed methods diagrams to depict points of integration, integration in the form of a joint display, and meta-inferences. They also highlighted that other important criteria include the skills of researchers, the use of philosophy, and the use of literature. There is also increasing recognition that evaluation occurs within a complex set of cultural dimensions, which mixed methods are ideally suited to address and could be featured in the future iteration of the GRAMMS [46]. Mixed methods research is increasingly collaborative, and efforts to advance collaboration in MMR have also had associated reporting criteria associated with it [47]. Furthermore, the criterion regarding limitations of one method related to the presence of the other method (criterion 5) is hardly present in most studies, as indicated by several methodological reviews, which may indicate that researchers do not know how to think about MMR limitations and tend to think about generic limitations of qualitative and quantitative research [8,35].

It is essential to close these gaps to leverage the true benefits of MMR in health research. Developing an updated GRAMMS guideline and checklist based on the Enhancing the Quality and Transparency of Health Research (EQUATOR) methodology (ie, involving a methodological review of quality reporting criteria in MMR as well as a modified Delphi process) is one way to close this gap and is essential to promote quality reporting of mixed methods studies in health research and interdisciplinary fields so that researchers, practitioners, decision makers, and other relevant knowledge users have access to high quality MMR study findings to inform practice and policy making. This proposal outlines the aim to develop the GRAMMS 2.0 guideline and checklist to improve the consistency, transparency, and quality of reporting of studies that use mixed methods in health research.

## Methods

### Study Design

The development of GRAMMS 2.0 will follow the established methodological framework for reporting guideline development, as outlined by the EQUATOR Network [48] and the Guidance for Developers of Health Research Reporting Guidelines [49]. The EQUATOR Network is an international initiative that aims to improve the quality of health research publications by promoting transparent and accurate reporting [48]. The Network’s website contains a comprehensive and up-to-date database of reporting guidelines relevant to health research. As part of this process, we have registered our GRAMMS 2.0 proposal on the EQUATOR Network website to ensure alignment with international standards and enhance transparency.

We will also adopt an integrated knowledge translation (iKT) approach, engaging a diverse panel that includes patient partners with experience in MMR, as well as researchers, journal editors, and funders. This panel will be guided by the Ontario Brain Institute’s “Ways Community Members Can Participate in the Stages of Research” framework [50] to ensure meaningful engagement throughout the project. To further support inclusive and respectful collaboration, we will use the reflective exercise titled “Strategy for Patient-Oriented Research Evidence Alliance Reflective EDI Exercise” to encourage open dialogue and understanding around equity, diversity, and inclusion topics [51]. These discussions will be conducted in a way that values diverse perspectives and promotes trust, respect, and inclusion. The iKT panel will play an active role in various stages of the project, including shaping the literature search strategy, reviewing preliminary findings, and planning how the final GRAMMS 2.0 guideline and checklist will be shared and applied. The Guidance for Reporting Involvement of Patients and Public (GRIPP2) will guide the reporting of our patient engagement efforts for the iKT panel as well as participation in study phases [52].

### Study Phases

#### Phase 1: Methodological Review of Quality Reporting Criteria in Mixed Methods Research

We will conduct a methodological review based on the guidelines for these types of reviews proposed in the literature [53-55] to identify and summarize existing literature on the quality of reporting of mixed methods research. This approach is well-suited for summarizing a broad range of methodological sources, including MMR systematic methodological reviews as well as discussion papers focused on quality reporting in MMR across various disciplines. The goal is to systematically identify and analyze key themes and proposed reporting criteria, providing a comprehensive overview of the current evidence in the field. Our review will specifically address the question: “What quality reporting criteria are proposed in the methodological literature on mixed methods?” This protocol paper will serve as the published protocol for the scoping review component of the study. To support transparency and accessibility, all review data will be made publicly available on the OSF. The completed PRISMA-ScR checklist is shown in Multimedia Appendix 1.

##### Selection Criteria and Search Strategy

We will apply the following inclusion criteria: (1) mixed methods methodological literature published in 2008 or later when the GRAMMS was first published; and (2) systematic, scoping, prevalence, or methodological reviews of empirical mixed methods studies examining the quality of reporting in those studies or discussion papers about quality reporting in MMR. We will also consider any existing MMR reporting tools. We will include any review and discussion paper regardless of the discipline or field that has examined the methodological reporting quality of mixed methods research studies, and the publication language. For reviews published in languages other than English, French, Spanish, and Finnish, DeepL [56] will be used to determine whether they meet the inclusion criteria. The use of DeepL for the initial screening of non-English publications may introduce minor screening inaccuracies due to translation limitations; however, we will mitigate this risk by having a fluent speaker of the respective language review the translation of any of these articles to ensure accuracy. We will exclude methodological literature that is not peer-reviewed, such as editorials, commentaries, mixed methods guides, books, and book chapters, to ensure that the included evidence has undergone scholarly peer review and presents sufficient methodological transparency for critical appraisal. However, to mitigate the risk of omitting foundational MMR guidance that may appear in seminal books or chapters, we will scan the reference lists of included reviews to identify any such works. If these sources are recurrently cited as influential, we will describe them narratively to contextualize the peer-reviewed evidence base. We will search the following electronic databases from 2008: Medline, CINAHL, Scopus, Embase, Cochrane, PsycINFO, ERIC, Web of Science, JSTOR, Sociological Abstracts, and ECONLit.

A librarian with expertise in knowledge synthesis will design a structured search strategy using both subject headings and relevant text words related to MMR, quality, reporting, and reviews. The search strategy will be peer-reviewed by another librarian using the Peer Review of Electronic Search Strategies (PRESS) checklist [57] as well as reviewed by our iKT panel. In addition, to capture relevant literature not indexed in these databases, we will spot-check key discipline-specific journals, including the *Journal of Mixed Methods Research* and the *International Journal of Multiple Research Approaches*. This approach ensures that foundational and highly relevant methodological articles are not missed.

##### Screening Process and Data Extraction

All screening (ie, levels 1 and 2) will occur in duplicate and independently. For level 1 screening, reviewers will screen the titles and abstracts for inclusion using the screening form. A pilot test of the level 1 screening form on a random sample of approximately 100 articles will be conducted to increase reliability. The descriptions of the eligibility criteria will be revised if necessary by the team or if a low agreement (ie, < 70%) is observed to improve the consistent application of the selection criteria. For level 2, the full text of potentially relevant articles will be collected and screened to determine final inclusion. A pilot test of the level 2 screening form will also be performed on approximately 1% of the articles, similar to the process for level 1 screening. Another reviewer knowledgeable in the research area will be available to resolve conflicts when necessary.

Extracted data will include the first author’s name, year of publication, number of studies included, types of mixed methods designs, time frame (in years) of the studies included in the article, first author’s institution’s country affiliation, type of review, geographical focus, population focus, the components of the GRAMMS addressed, components of the quality reporting not addressed by the GRAMMS, primary findings related to reporting quality, limitations of each review, and recommendations for reporting of mixed methods studies. Additional categories will likely be identified through the completion of the search and discussions with the research team, including the iKT Panel. The data extraction form will be pilot-tested and refined with at least two research team members. Like the level 1 and 2 screening processes, data extraction will occur in duplicate and independently. Discussion or the involvement of another reviewer will resolve disagreements. Covidence will be used to manage the records and data throughout the review.

##### Quality Assessment

Two reviewers will use the Critical Appraisal Tool for Health Promotion and Prevention Reviews (CAT HPPR) to assess all systematic, rapid, and scoping reviews. This tool is well-established to evaluate the quality of reviews and meta-analyses and evaluates the reviews in terms of review question, search strategy, risk of bias assessment, data extraction and synthesis, and reporting quality [58]. Although the tool is relatively new and was not specifically designed for methodological or scoping reviews, it was selected because its domains align with core quality criteria relevant to assessing the transparency, rigor, and reproducibility of review processes. For scoping reviews, which are not typically subject to formal critical appraisal, the CAT-HPPR will be applied in a descriptive rather than evaluative manner to identify methodological strengths and gaps. Any adaptations or interpretive decisions will be documented and reported to ensure transparency.

The methodological review is expected to begin in Fall 2025 and is anticipated to conclude within one year. The results of this methodological review will be used to inform the surveys implemented in Phase 2, as well as the draft GRAMMS 2.0 guideline. The review’s findings will help identify which items need to remain, be modified, or be added to the GRAMMS 2.0. Findings will be published in a peer-reviewed publication*,* as well as disseminated via a newsletter and webinar to promote engagement for Phase 2.

#### Phase 2: Modified Delphi Process to Prioritize Items for the GRAMMS 2.0 Guideline

To prioritize the items for inclusion in the GRAMMS 2.0 guideline, we will use a modified Delphi process, a well-established method for building consensus in the development of reporting guidelines [49]. This process involves a series of surveys administered over multiple rounds to gather expert opinions and refine the list of items based on collective feedback. Participants are typically asked to rate or rank the importance of various items, with each round helping to narrow the list and move toward consensus. The modified Delphi differs from the conventional Delphi process in that it begins the process with preselected items drawn from earlier work, rather than using the experts to brainstorm on a particular subject [59]. In line with the approach used to develop other reporting guidelines [60,61], we anticipate conducting 3 rounds of the Delphi process. Each round of the Delphi process will identify items with strong agreement and reduce the number of items based on participant responses and expert consensus. A virtual consensus meeting will then be held following the Delphi. The ACcurate COnsensus Reporting Document (ACCORD) will be used to report the findings from the modified Delphi approach and consensus meeting [62].

##### Identification of Participants for the Modified Delphi Process and Virtual Consensus Meeting

An international, interdisciplinary, and diverse group of patients, funders, policymakers, researchers, and journal editors with experience or expertise in MMR will be invited to participate in the modified Delphi process. Participants will be identified through various sources, including authors of published MMR studies, authors of mixed methods books, instructors of mixed methods courses, and leadership and members of the Mixed Methods International Research Association. We will also use social media outlets, including X, Facebook, Threads, Blue Sky, and LinkedIn. Recognizing and addressing the appropriate use of sex and gender terms in reporting guidelines has been noted as an important gap [63]. As such, we are committed to ensuring that GRAMMS 2.0 reflects best practices in this area by including perspectives from a broad range of groups and ensuring proper representation throughout the Delphi process [63].

We will use a purposive and snowball sampling strategy to achieve the recommended minimum sample size of 100-150, consistent with other reporting guideline initiatives [60]. Potential expert participants will be invited to participate by email, including an explanatory statement about the initiative. Participants who may have challenges accessing technology will be provided with the means to participate, for example, by conducting surveys over the phone, through Zoom, or by mailing the surveys.

##### Survey and Administration for Rounds 1-3

For Round 1, the survey will be based on the results of the proposed methodological review, as described above. The initial list of GRAMMS 2.0 guideline items and the survey will be reviewed with the research team; they will be pilot-tested for content and clarity and revised before dissemination. Only those participants who complete the survey in Round 1 will be invited to participate in the survey in Round 2. Similarly, participants must complete the Round 2 survey to be invited for the Round 3 survey. Demographic and descriptive information about the participants will be collected (eg, sex, gender identity, race, country of residence, expert participant type, career stage, etc) to ensure a diverse sample. However, these items will be optional as some people may feel uncomfortable reporting them.

Survey administration will last 4 weeks per round, with reminder emails sent every 7 days after the initial invitation to optimize participation. For all rounds, participants will be asked to rate their agreement with the inclusion of each of the proposed guideline items using a 5-point Likert scale (from 1=“strongly disagree” to 5=“strongly agree”). Surveys will be administered in English. Each survey item will include an optional text box for participants to provide comments, including suggestions for any additions, deletions, aggregation, and refinement of items (eg, there will be opportunities to retain items in a modified form).

##### Survey Analysis

Demographic and descriptive information of the participants will be analyzed with proportions expressed as percentages (eg, sex, gender identity, country of residence, race, knowledge user type, career stage, etc). For all 3 rounds, a threshold of 70% agreement will be applied for each guideline item to indicate a consensus among the expert participants. That is, at least 70% of participants must mostly or entirely agree (ie, values of 4 or 5 on the Likert scale) with the inclusion or exclusion of the guideline item as part of GRAMMS 2.0. This threshold is consistent with developing other reporting guidelines, such as the PRISMA-ScR (Preferred Reporting Items for Systematic reviews and Meta-Analyses extension for Scoping Reviews) [60]. If less than 70% of participants mostly or entirely agree with the item’s inclusion or exclusion, it will be considered discrepant. Persistent discrepant items will be brought forward for structured discussion during the consensus meeting. Where possible, we will investigate whether item agreement differs by sex, gender, other aspects of diversity, and knowledge user type (ie, chi-square analysis).

When applicable, content analysis [64] will be conducted on the comments. These results will be summarized and used to inform subsequent surveys. Furthermore, for the Round 2 and 3 surveys, participants will be given their results and the overall group distribution, median, and IQR from the previous survey rounds (ie, Round 1 and 2 surveys). These data will promote transparency and encourage careful reflection on items where consensus has not been reached. In addition, as part of this exercise, in the Round 2 and 3 surveys, we will ask participants to respond to the question, “After reviewing your survey results for this item (ie, each discrepant item), please comment on why you rated this item the way you did.” Content analysis [64] will be conducted on these comments.

##### Consensus Meeting

Two half-day virtual consensus meetings will be held after completing the 3 rounds of surveys. Approximately 25-30 expert participants who completed the survey in Rounds 1-3 will be invited to participate in the consensus meeting. Consideration will be given to include 25-30 expert participants with a diverse range of characteristics, for example, sex, gender, country, expertise in MMR, that is, mixed methods trainees, as well as senior-career mixed methods researchers, and discipline/subdiscipline. A skilled facilitator will lead the consensus meeting, generate meeting minutes, and compile the discussion to produce a summary of results. The facilitator will be an independent moderator in order to ensure balanced participation. A summary of the results from Round 3 and any accompanying recommendations will be presented. These data will be shared to generate and inform the discussion on any critical discrepant items (ie, key issues and considerations relating to both items and wording). Participants will be asked to elaborate on why they rated a discrepant item the way they did. To mitigate potential dominance of academic or research voices, the iKT panel will co-develop a plan to share with the facilitator to promote equitable engagement during the consensus meeting. This plan will specify how discussions, feedback, and decision-making will occur and will outline concrete facilitation strategies to ensure balanced participation and prevent overrepresentation of any single expert group.

A draft version of the GRAMMS 2.0 guideline will be created by the research team with the iKT panel and then presented for discussion at the consensus meeting. The expert participants will discuss these proposed items and reach a consensus on which items should be included in the final version of GRAMMS 2.0 and their specific wording. As part of this exercise, we will also discuss the potential recommendation of a core set of items of the guideline and checklist (including a discussion of how each guideline item will be linked to a checklist item) and the possible weighting of items, if appropriate/feasible. As such, we will consider developing a short and long version of GRAMMS 2.0.

Furthermore, we will discuss how the GRAMMS 2.0 guideline and checklist can promote diverse perspectives and the reporting of these diverse perspectives (eg, use of culturally specific paradigms). We will also use the consensus meeting to discuss the overall dissemination of our results. We will conduct a comprehensive recording (ie, digital recording as well as note-taking) of all the discussions related to the GRAMMS 2.0 guideline and checklist development, including providing a record of the decisions made during the meeting. Content analysis, performed in duplicate, will be used to analyze the digital recording of the meeting [64]. Notes taken at the meeting will be used to supplement the recordings and aid the analysis. After the consensus meeting, the GRAMMS 2.0 guideline and checklist will be distributed to the expert participants to ensure they reflect the decisions made.

##### Finalization of GRAMMS 2.0

The final GRAMMS 2.0 guideline and checklist will be tested with approximately 15 researchers/scientists, decision-makers, and students by applying it to at least one of their MMR for relevance and feasibility (ie, persons not involved in the Delphi). Following the testing, the research team with iKT panel members will meet in person to discuss and finalize GRAMMS 2.0.

The final GRAMMS 2.0 guideline and accompanying checklist will also be posted on the EQUATOR Network’s website. To promote the uptake of GRAMMS 2.0, we will create a 2-minute video outlining how to operationalize each item, webinars for key organizations that conduct MMR, and a 1-page infographic. We may publish the GRAMMS 2.0 guideline and checklist in multiple journals simultaneously to promote uptake and dissemination. The first author’s (SM’s) website will provide updates on the project and promote dissemination. We will endeavor to locate and email authors who have registered a mixed methods study to disseminate GRAMMS 2.0. We will also explore automated tools to promote the uptake of GRAMMS 2.0, including an email system in which authors would be sent the GRAMMS 2.0 upon registering or publishing their mixed methods protocol. Penelope.ai is another online tool that could be leveraged, which verifies manuscripts, for example, provides comprehensive reporting, and provides authors with feedback. It should be noted that these specific strategies have been adopted with the PRISMA-ScR [60] guideline and have contributed to it being cited >23,000 times since 2018. Additionally, we will share the GRAMMS 2.0 guideline and checklist widely within our networks.

### Ethical Considerations

Research Ethics Board approval for this component of the study will be sought at Holland Bloorview Kids Rehabilitation Hospital (ie, the lead investigator’s primary institution).

## Results

This work is supported by a Project Grant from the Canadian Institutes of Health Research (CIHR) received in April 2025 (202409PJT-527857-HS1-CEAS-178733; Multimedia Appendix 2). An iKT panel, including patient partners with experience in mixed methods research, as well as researchers, journal editors, and funders, will be established to contribute to various stages of the project. We anticipate that Phase 1 of the study will begin in Fall 2025 and be completed within one year. Ethics approval for Phase 1 is not required as it will not involve collecting or using data from participants. Ethics approval for Phase 2 of the study will be sought in Fall 2026. Phase 2, including the modified Delphi process and consensus meeting, is anticipated to be completed by 2027.

## Discussion

### Principal Findings

The collective results of this project will lead to the development of a ready-to-implement GRAMMS 2.0 guideline that outlines a minimum set of items to include in health research using mixed methods.

### Challenges and Mitigating Strategies

Recruiting diverse participants for our modified Delphi process may be difficult. To address this challenge, we will leverage our association with and support of the Mixed Methods International Research Association and review our recruitment material across a range of demographic and geographical diversity biases. We will also invite mixed methods researchers who have worked with or are known to the research team. To ensure that members of the modified Delphi process and consensus panel are not limited in their participation due to a lack of access to devices and technology (eg, individuals from rural and remote communities), we will mail paper copies of the surveys on request. Finally, we also acknowledge that holding half-day consensus meetings may be challenging for some participants. Organizing meetings across time zones will also be challenging. We will ask our participants about their preferences regarding scheduling and timing, and, if preferred, hold the consensus meeting for a few hours, but over a couple of days/weeks. We will also make accommodations for those with vision and hearing impairments (eg, closed captioning).

### Conclusions

The GRAMMS 2.0 guideline will improve the consistency, transparency, and quality of reporting of mixed methods studies by researchers and multiple knowledge users in policy and practice and across different disciplines. A key strength of this work is leveraging the existing GRAMMS reporting guideline as well as the expertise of its developer (AOC). Peer reviewers and editors will also use the GRAMMS 2.0 to improve the review of manuscripts involving MMR. Ultimately, the GRAMMS 2.0 guideline will increase the clarity and credibility of MMR findings, increasing their potential for practical application and contributing to the continued advancement of mixed methods methodology. In turn, this will facilitate more effective use of new results in mixed methods in health research and maximize the return on investment.

## Appendix

Multimedia Appendix 1 PRISMA-ScR (Preferred Reporting Items for Systematic reviews and Meta-Analyses extension for Scoping Reviews) checklist.

Multimedia Appendix 2

## Abbreviations

ACCORD: ACcurate COnsensus Reporting Document
CAT HPPR: Critical Appraisal Tool for Health Promotion and Prevention Reviews
EQUATOR: Enhancing the Quality and Transparency of Health Research
GRAMMS: Good Reporting of a Mixed Methods Study
GRIPP2: Guidance for Reporting Involvement of Patients and Public
iKT: integrated knowledge translation
MMR: mixed methods research
PRESS: Peer Review of Electronic Search Strategies
PRISMA-ScR: Preferred Reporting Items for Systematic reviews and Meta-Analyses extension for Scoping Reviews

## Data Availability

The data collected during this study are available from the corresponding author upon reasonable request.
