# Supplementary material for: Updating the Good Reporting of a Mixed Methods Study (GRAMMS) Reporting Guidelines: Protocol for a Methodological Review and Modified Delphi Process
Source: JMIR Res Protoc. 2026 Mar 17;15:e82364. doi: 10.2196/82364 (PMC13040166; doi:10.2196/82364)
Supplement: Multimedia Appendix 2 [file resprot_v15i1e82364_app2.pdf]

**Review Type / Type d'évaluation:**	Reviewer 1 / Évaluateur 1
**Name of Applicant / Nom du chercheur:**	Munce, Sarah
**Application No. / Numéro de demande:**	527857
**Agency / Agence:**	CIHR/IRSC
**Competition / Concours:**	Project Grant/Subvention Projet
**Committee / Comité:**	Health Services Evaluation & Interventions Research/Recherche en interventions et en évaluation dans les services de santé
**Title / Titre:**	Updating the Good Reporting of Mixed Methods Study (GRAMMS) Reporting Guidelines

Adjudication Criteria/Critères de sélection

Initial Score/Cote Initiale: 4.4

Top/Bottom Selection/Groupe supérieur/inférieur

- (●) **Top/Groupe supérieur**
- ( ) **Bottom/Groupe inférieur**

**Review Type / Type d'évaluation:**	Reviewer 1 / Évaluateur 1
**Name of Applicant / Nom du chercheur:**	Munce, Sarah
**Application No. / Numéro de demande:**	527857
**Agency / Agence:**	CIHR/IRSC
**Competition / Concours:**	Project Grant/Subvention Projet
**Committee / Comité:**	Health Services Evaluation & Interventions Research/Recherche en interventions et en évaluation dans les services de santé
**Title / Titre:**	Updating the Good Reporting of Mixed Methods Study (GRAMMS) Reporting Guidelines

Summary of Application/Résumé de la demande:

The purpose of this project is to develop updated GRAMMS 2.0 guideline and checklist to improve consistency, transparency, and quality of reporting of studies that use mixed methods in health services research. Project will be conducted in two phases using typical methods to develop reporting guidelines including a review of the literature on quality reporting in the mixed methods literature followed by rounds of surveys and a consensus meeting to finalize the new GRAMMS guidelines.

Aim is to develop the GRAMMS 2.0 guideline and checklist to improve consistency, transparency, and quality of reporting of studies that use mixed methods in health services research. Objectives are to: 1) examine the mixed methods methodological literature and identify relevant reporting quality criteria for inclusion in GRAMMS 2.0 and 2) use the results of the methodological review to prioritize the components of the updated GRAMMS 2.0 using a modified Delphi approach and consensus meeting.

Research question is: What quality reporting criteria are proposed in mixed methods methodological literature?

Plan to use an integrated knowledge translation (iKT) approach including a panel of patient partners with mixed method study experience, researchers, journal editors, and funders. The iKT Panel activities will be guided by the Ontario Brain Institute's "Ways Community Members Can Participate in the Stages of Research" framework. Plan to use the "SPOREA Reflective EDI Exercise" to promote dialogue and understanding around equity, diversity, and inclusion (EDI) topics amongst all iKT Panel members.

For Round 1, the survey will be based on the results of the proposed methodological review. The initial list of GRAMMS 2.0 guideline items and the survey will be reviewed with the research team; pilot-tested for content and clarity; and revised before dissemination. Only those participants who complete the survey in Round 1 will be invited to participate in the survey in Round 2. Similarly, participants must complete the Round 2 survey to be invited for the Round 3 survey. Demographic and descriptive information about the participants will be collected.

**Review Type / Type d'évaluation:**	Reviewer 1 / Évaluateur 1
**Name of Applicant / Nom du chercheur:**	Munce, Sarah
**Application No. / Numéro de demande:**	527857
**Agency / Agence:**	CIHR/IRSC
**Competition / Concours:**	Project Grant/Subvention Projet
**Committee / Comité:**	Health Services Evaluation & Interventions Research/Recherche en interventions et en évaluation dans les services de santé
**Title / Titre:**	Updating the Good Reporting of Mixed Methods Study (GRAMMS) Reporting Guidelines

Strengths and Weaknesses/Forces et faiblesses:

Strengths

Addresses a much needed update. Have responded to reviewer feedback. They have provided a more compelling rationale for the GRAMMS update, the main one being to keep up with advancement in methods for integration of quant and qual results using methods such as joint displays and meta-inferences. The 2008 methodology which the guideline is based did not incorporate this.

Will register the protocol for the review with the Open Science Framework.

GRAMMS 2.0 guideline items will be prioritized using a modified Delphi process, a consensus building process. published in a peer-reviewed journal (e.g., BMJ Open).

Will leverage team's association with the Mixed Methods International Research Association (MMIRA), including Collaborator Guetterman's role as the MMIRA President to access diverse members representing a range of countries and MMR Chapters such as Oceania, China, Latin America, Caribbean, Japan, and Francophone.

Have added details on how the mixed methods field has advanced since 2008 and areas that current GRAMMS does not address such as cross-cultural mixed methods studies, sex-gender-based analysis, and considerations of equity and diversity reporting in mixed methods research.

Have also attended to how patient partners will be involved in the iKT panel noting that these activities will be guided by the Ontario Brain Institutes' Ways Community Members can Participate in Stages of Research framework. Have highlighted how team members, for example, Munce, Younas, Poth, Guetterman, Fabregues, Hong, Creswell are associated with the Mixed Methods International Research Association (MMIRA), noting that Collaborator, Guetterman, is the President of MMIRA. These associations will facilitate access to diverse members representing a range of countries and cultures, for example in Oceania, China, Latin America, Caribbean, Japan, and Francophone communities.

Have identified how they will select the 25-30 expert participants for the Consensus meeting to include diverse characteristics e.g., sex, gender, country, expertise in mixed methods research.

Have further developed KT processes.

**Review Type / Type d'évaluation:**	Reviewer 1 / Évaluateur 1
**Name of Applicant / Nom du chercheur:**	Munce, Sarah
**Application No. / Numéro de demande:**	527857
**Agency / Agence:**	CIHR/IRSC
**Competition / Concours:**	Project Grant/Subvention Projet
**Committee / Comité:**	Health Services Evaluation & Interventions Research/Recherche en interventions et en évaluation dans les services de santé
**Title / Titre:**	Updating the Good Reporting of Mixed Methods Study (GRAMMS) Reporting Guidelines

Budget Recommendation/Recommandation budgétaire:

In response to prior reviewer comments re the budget, the researchers have decided to hold the consensus meeting virtually and an in-person team meeting with core research team. This reduces the budget by 255K and shortens the project to 2 years.

Please indicate your appraisal of the integration of sex as a biological variable as a strength, weakness, or not applicable to the proposal./Prière de sélectionner une option pour donner votre évaluation de l'intégration du sexe comme variable biologique en tant que point fort ou point faible de la proposition, ou en tant qu'élément non applicable à la proposition.

- (●) **Strength/Point fort**
- ( ) **Weakness/Point faible**
- ( ) **Not applicable/Non applicable**

Please indicate your appraisal of the integration of gender as a socio-cultural determinant of health as a strength, weakness, or not applicable to the proposal./Prière de sélectionner une option pour donner votre évaluation de l'intégration du genre comme déterminant socioculturel de la santé en tant que point fort ou point faible de la proposition, ou en tant qu'élément non applicable à la proposition.

- (●) **Strength/Point fort**
- ( ) **Weakness/Point faible**
- ( ) **Not applicable/Non applicable**

**Review Type / Type d'évaluation:**	Reviewer 1 / Évaluateur 1
**Name of Applicant / Nom du chercheur:**	Munce, Sarah
**Application No. / Numéro de demande:**	527857
**Agency / Agence:**	CIHR/IRSC
**Competition / Concours:**	Project Grant/Subvention Projet
**Committee / Comité:**	Health Services Evaluation & Interventions Research/Recherche en interventions et en évaluation dans les services de santé
**Title / Titre:**	Updating the Good Reporting of Mixed Methods Study (GRAMMS) Reporting Guidelines

Sex and/or Gender Considerations/Notions de sexe et/ou de genre:

Will ensure that GRAMMS 2.0 adheres to criteria for correct use of sex and gender terms in reporting guidelines. Delphi process will ensure representation of different groups e.g., various sex, gender, and cultural groups. Analysis, where possible, will include whether item agreement differs by sex, gender, other aspects of diversity, and knowledge user type (i.e., Chi-Square analysis).

**Review Type / Type d'évaluation:**	Reviewer 2 / Évaluateur 2
**Name of Applicant / Nom du chercheur:**	Munce, Sarah
**Application No. / Numéro de demande:**	527857
**Agency / Agence:**	CIHR/IRSC
**Competition / Concours:**	Project Grant/Subvention Projet
**Committee / Comité:**	Health Services Evaluation & Interventions Research/Recherche en interventions et en évaluation dans les services de santé
**Title / Titre:**	Updating the Good Reporting of Mixed Methods Study (GRAMMS) Reporting Guidelines

Adjudication Criteria/Critères de sélection

Initial Score/Cote Initiale: 4.0

Top/Bottom Selection/Groupe supérieur/inférieur

- (●) **Top/Groupe supérieur**
- ( ) **Bottom/Groupe inférieur**

**Review Type / Type d'évaluation:**	Reviewer 2 / Évaluateur 2
**Name of Applicant / Nom du chercheur:**	Munce, Sarah
**Application No. / Numéro de demande:**	527857
**Agency / Agence:**	CIHR/IRSC
**Competition / Concours:**	Project Grant/Subvention Projet
**Committee / Comité:**	Health Services Evaluation & Interventions Research/Recherche en interventions et en évaluation dans les services de santé
**Title / Titre:**	Updating the Good Reporting of Mixed Methods Study (GRAMMS) Reporting Guidelines

Summary of Application/Résumé de la demande:

This proposal seeks to update the Good Reporting of a Mixed Methods Study (GRAMMS) guidelines, which are over 15 years old. The updated GRAMMS 2.0 aims to improve the consistency, transparency, and quality of reporting in mixed methods health services research. The project will involve a methodological review of existing literature and a modified Delphi approach to prioritize components of the updated guidelines.

**Review Type / Type d'évaluation:**	Reviewer 2 / Évaluateur 2
**Name of Applicant / Nom du chercheur:**	Munce, Sarah
**Application No. / Numéro de demande:**	527857
**Agency / Agence:**	CIHR/IRSC
**Competition / Concours:**	Project Grant/Subvention Projet
**Committee / Comité:**	Health Services Evaluation & Interventions Research/Recherche en interventions et en évaluation dans les services de santé
**Title / Titre:**	Updating the Good Reporting of Mixed Methods Study (GRAMMS) Reporting Guidelines

Strengths and Weaknesses/Forces et faiblesses:

Strengths

Clear and focused objectives with a defined output.

Adheres to established guidelines for developing reporting guidelines.

Employs an appropriate modified Delphi approach.

Diverse team with representation from across the country.

Well-considered mitigation strategies for potential challenges.

Weaknesses

May face challenges in achieving broad international acceptance and adoption of the updated guidelines.

The potential impact of rapidly emerging AI technologies on current practices and guidelines should be considered, although this may be addressed through the Delphi process.

**Review Type / Type d'évaluation:**	Reviewer 2 / Évaluateur 2
**Name of Applicant / Nom du chercheur:**	Munce, Sarah
**Application No. / Numéro de demande:**	527857
**Agency / Agence:**	CIHR/IRSC
**Competition / Concours:**	Project Grant/Subvention Projet
**Committee / Comité:**	Health Services Evaluation & Interventions Research/Recherche en interventions et en évaluation dans les services de santé
**Title / Titre:**	Updating the Good Reporting of Mixed Methods Study (GRAMMS) Reporting Guidelines

Budget Recommendation/Recommandation budgétaire:

Budget on high side for activities

Please indicate your appraisal of the integration of sex as a biological variable as a strength, weakness, or not applicable to the proposal./Prière de sélectionner une option pour donner votre évaluation de l'intégration du sexe comme variable biologique en tant que point fort ou point faible de la proposition, ou en tant qu'élément non applicable à la proposition.

- (●) **Strength/Point fort**
- ( ) **Weakness/Point faible**
- ( ) **Not applicable/Non applicable**

Please indicate your appraisal of the integration of gender as a socio-cultural determinant of health as a strength, weakness, or not applicable to the proposal./Prière de sélectionner une option pour donner votre évaluation de l'intégration du genre comme déterminant socioculturel de la santé en tant que point fort ou point faible de la proposition, ou en tant qu'élément non applicable à la proposition.

- (●) **Strength/Point fort**
- ( ) **Weakness/Point faible**
- ( ) **Not applicable/Non applicable**

**Review Type / Type d'évaluation:**	Reviewer 2 / Évaluateur 2
**Name of Applicant / Nom du chercheur:**	Munce, Sarah
**Application No. / Numéro de demande:**	527857
**Agency / Agence:**	CIHR/IRSC
**Competition / Concours:**	Project Grant/Subvention Projet
**Committee / Comité:**	Health Services Evaluation & Interventions Research/Recherche en interventions et en évaluation dans les services de santé
**Title / Titre:**	Updating the Good Reporting of Mixed Methods Study (GRAMMS) Reporting Guidelines

Sex and/or Gender Considerations/Notions de sexe et/ou de genre:

Sex and gender appropriately considered.

**Review Type / Type d'évaluation:**	Reviewer 3 / Évaluateur 3
**Name of Applicant / Nom du chercheur:**	Munce, Sarah
**Application No. / Numéro de demande:**	527857
**Agency / Agence:**	CIHR/IRSC
**Competition / Concours:**	Project Grant/Subvention Projet
**Committee / Comité:**	Health Services Evaluation & Interventions Research/Recherche en interventions et en évaluation dans les services de santé
**Title / Titre:**	Updating the Good Reporting of Mixed Methods Study (GRAMMS) Reporting Guidelines

Adjudication Criteria/Critères de sélection

Initial Score/Cote Initiale: 4.0

Top/Bottom Selection/Groupe supérieur/inférieur

- (●) **Top/Groupe supérieur**
- ( ) **Bottom/Groupe inférieur**

**Review Type / Type d'évaluation:**	Reviewer 3 / Évaluateur 3
**Name of Applicant / Nom du chercheur:**	Munce, Sarah
**Application No. / Numéro de demande:**	527857
**Agency / Agence:**	CIHR/IRSC
**Competition / Concours:**	Project Grant/Subvention Projet
**Committee / Comité:**	Health Services Evaluation & Interventions Research/Recherche en interventions et en évaluation dans les services de santé
**Title / Titre:**	Updating the Good Reporting of Mixed Methods Study (GRAMMS) Reporting Guidelines

Summary of Application/Résumé de la demande:

The applicants propose to develop a Good Reporting of a Mixed Methods Study 2.0 (GRAMMS 2.0) guideline, intended to improve quality, consistency and transparency in mixed methods research reporting. (The proposal notes the somewhat distant release of the original GRAMMS guidelines in 2008, which would be updated through this project to address advances in the field.) The proposed project includes two objectives: (1) to examine methodological literature on mixed methods research to identify potential quality criteria for a proposed new reporting guideline, and (2) to prioritize quality criteria as components of the updated guideline through a modified Delphi approach and consensus meeting.

**Review Type / Type d'évaluation:**	Reviewer 3 / Évaluateur 3
**Name of Applicant / Nom du chercheur:**	Munce, Sarah
**Application No. / Numéro de demande:**	527857
**Agency / Agence:**	CIHR/IRSC
**Competition / Concours:**	Project Grant/Subvention Projet
**Committee / Comité:**	Health Services Evaluation & Interventions Research/Recherche en interventions et en évaluation dans les services de santé
**Title / Titre:**	Updating the Good Reporting of Mixed Methods Study (GRAMMS) Reporting Guidelines

Strengths and Weaknesses/Forces et faiblesses:

Strengths

A rationale for developing an updated reporting guideline for mixed methods research is well described, with a thorough account of advances in the field since the publication of the existing guideline.

Planned methods for both phases of the project are clearly described, including the search strategy, selection criteria and screening of methodological literature, data extraction and quality assessment as well as the proposed modified Dephi process and virtual consensus meeting.

A detailed knowledge translation plan is provided, supported by leadership roles of multiple team members in relevant organizations and communities of practice. The application note previous success in knowledge translation by team members on other reporting guidelines.

Consideration is given to recruiting and supporting diverse participants in the modified Delphi process and consensus meeting.

The Principal Applicant has an extensive track record of funding and publication in mixed methods research. As well, their record of publication, teaching, and leadership in their scientific community reflect the methodological expertise appropriate to the project aims. Their current program of funded research includes development of another reporting guideline.

The application team includes senior researchers in the proposal topic, as well as a knowledge user with whom the Principal Applicant is an ongoing research partner (who has provided a letter of support as a youth engaged in mixed methods research).

Budget Recommendation/Recommandation budgétaire:

Recommended reduction:

$11,250 in honoraria for experts completing surveys for the modified Delphi process surveys seems high. (150 participants X 3 surveys X $25/survey.) Noting that the planned participants will be mainly “funders, policymakers, researchers and journal editors with experience/expertise in MMR,” it seems reasonable to apply other means to promote the participation of these professionals.

Please indicate your appraisal of the integration of sex as a biological variable as a strength, weakness, or not applicable to the proposal./Prière de sélectionner une option pour donner votre évaluation de l'intégration du sexe comme variable biologique en tant que point fort ou point faible de la proposition, ou en tant qu'élément non applicable à la proposition.

- (●) **Strength/Point fort**
- ( ) **Weakness/Point faible**
- ( ) **Not applicable/Non applicable**

Please indicate your appraisal of the integration of gender as a socio-cultural determinant of health as a strength, weakness, or not applicable to the proposal./Prière de sélectionner une option pour donner votre évaluation de l'intégration du genre comme déterminant socioculturel de la santé en tant que point fort ou point faible de la proposition, ou en tant qu'élément non applicable à la proposition.

- (●) **Strength/Point fort**
- ( ) **Weakness/Point faible**
- ( ) **Not applicable/Non applicable**

**Review Type / Type d'évaluation:**	Reviewer 3 / Évaluateur 3
**Name of Applicant / Nom du chercheur:**	Munce, Sarah
**Application No. / Numéro de demande:**	527857
**Agency / Agence:**	CIHR/IRSC
**Competition / Concours:**	Project Grant/Subvention Projet
**Committee / Comité:**	Health Services Evaluation & Interventions Research/Recherche en interventions et en évaluation dans les services de santé
**Title / Titre:**	Updating the Good Reporting of Mixed Methods Study (GRAMMS) Reporting Guidelines

Sex and/or Gender Considerations/Notions de sexe et/ou de genre:

Consideration of sex and gender is described in relation to the project methodology and for the principal output.

Review Type/Type d'évaluation: SO Notes /Notes de l'agent scientifique
Name of Applicant/Nom du chercheur: Munce, Sarah Elizabeth
Application No./Numéro de demande: 527857
Agency/Agence: CIHR/IRSC
Competition/Concours: 2024-09-11 Project Grant/Subvention Projet
Committee/Comité: Health Services Evaluation & Interventions Research/Recherche en interventions et en évaluation dans les services de santé
Title/Titre: Updating the Good Reporting of Mixed Methods Study (GRAMMS) Reporting Guidelines

---

Assessment/Évaluation:

Strengths (including SGBA considerations):

This is a resubmission of a project that was seen as important.
The applicants have responded well to previous reviewer comments.
The team has a track record in the field of developing research methodology guidelines
The applicants have described a compelling rationale for the need for the update around mixing qual and quant results
Addressing how the field has advanced since 2008 e.g. cross-cultural, EDIA, sex and gender was seen as a strength
The outcome of the grant has the potential to impactthe quality of mixed-methods research
The prioritization exercise of items was seen as a strength
The application is thoughtful on recruitment of diverse perspectives
It was seen as a strength that the team leveragesrelationships withan international mixed methods organization- this will enable recruitment that is feasible and results that are generalizable and broadly applicable
Integrating patient partners will be guided by anapproach based on a strong framework on engagement
The team is experienced and known in the mixed methods field tofacilitate participant recruitment
The research activities appear feasibleand activities are clearly described
There is a thoughtful approach to knowledge user engagement e.g. including a knowledge user who is engaged with youth, with whom the NPI has worked.

Weaknesses (including SGBA considerations):

The team did not address how AI may impact relevance in the future (although the Delphi may elicit information on this)

SGBA – while some details are provided, the approach could be more specific.

Budget:

No concerns noted

****

**Review Type/Type d'évaluation:**	SO Notes /Notes de l'agent scientifique
**Name of Applicant/Nom du chercheur:**	Munce, Sarah Elizabeth
**Application No./Numéro de demande:**	527857
**Agency/Agence:**	CIHR/IRSC
**Competition/Concours:**	2024-09-11 Project Grant/Subvention Projet
**Committee/Comité:**	Health Services Evaluation & Interventions Research/Recherche en interventions et en évaluation dans les services de santé
**Title/Titre:**	Updating the Good Reporting of Mixed Methods Study (GRAMMS) Reporting Guidelines

Assessment/Évaluation:

Note: The final rating of the application, provided in the Notice of Recommendation (NOR) and Notice of Decision (NOD), is the averaged rating of the peer review committee members following the discussion of the application during the committee meeting, and therefore may differ from the ratings provided by the assigned reviewers in their respective reviews.

Remarque : La cote définitive de la demande, qui apparaît dans l’avis de recommandation et l’avis de décision, représente la moyenne des cotes accordées par les membres du comité d’évaluation par les pairs après avoir débattu de la demande à la réunion du comité. Elle peut donc différer de celle donnée par les évaluateurs dans leur évaluation respective.
